# Analysis of *Aspergillus* spp. Isolates According to Temporal–Spatial, Sociodemographic, and Clinical Variables—Microsatellite Typing of Clinical and Environmental Samples of *Aspergillus fumigatus* in a University Hospital in Sao Paulo, Brazil

**DOI:** 10.1111/myc.70126

**Published:** 2026-01-17

**Authors:** Claudia de Abreu Fonseca, Ricardo Araujo, Vivian Caso Coelho, Carlos Henrique Camargo, Marcello Mihailenko Chaves Magri, Adriana Lopes Motta, Marina Farrel Côrtes, Ana Carolina Mamana, Marjorie Vieira Batista, Daniel Valério da Silva Moreira, Ana Paula Croce, Mauro Cintra Giudice, André Nathan Costa, Sergio Eduardo Demarzo, Alexandra Gomes dos Santos, Thais Guimarães, Vera Lucia Teixeira de Freitas, Sílvia Figueiredo Costa, Maria Aparecida Shikanai Yasuda

**Affiliations:** ^1^ Departamento de Infectologia e Medicina Tropical, Faculdade de Medicina, FMUSP Universidade de Sao Paulo Sao Paulo Brazil; ^2^ Laboratorio de Investigacao Medica em Protozoologia, Bacteriologia e Resistencia Antimicrobiana (LIM 49), Hospital das Clinicas, Faculdade de Medicina Universidade de São Paulo Sao Paulo Brazil; ^3^ Divisao de Moléstias Infecciosas e Parasitarias do Hospital das Clinicas, HCFMUSP, Faculdade de Medicina Universidade de Sao Paulo Sao Paulo Brazil; ^4^ Laboratorio de Investigacao Medica em Imunologia (LIM 48), Hospital das Clinicas, HCFMUSP, Faculdade de Medicina Universidade de Sao Paulo Sao Paulo Brazil; ^5^ INEB – Instituto de Engenharia Biomedica Universidade do Porto Porto Portugal; ^6^ i3S – Instituto de Investigação e Inovação em Saúde Universidade do Porto Porto Portugal; ^7^ Núcleo de Doencas Entéricas e Infecções Por Patógenos Especiais – Instituto Adolfo Lutz Sao Paulo Brazil; ^8^ Laboratorio de Investigacao Medica em Microbiologia – Divisao Do Laboratorio Central (LIM 03) do Hospital das Clinicas, HCFMUSP, Faculdade de Medicina, Universidade de Sao Paulo Sao Paulo Brazil; ^9^ Laboratorio de Investigacao Medica em Micologia (LIM 53) do Hospital das Clinicas HCFMUSP, Faculdade de Medicina Universidade de Sao Paulo Sao Paulo Brazil; ^10^ Divisao de Pneumologia, Instituto Do Coracao, Hospital das Clinicas, Faculdade de Medicinada Universidade de Sao Paulo Sao Paulo Brazil; ^11^ Servico de Endoscopia Respiratoria – Divisao de Pneumologia – Instituto Do Coracao (InCor) – Hospital das Clinicas, HCFMUSP, Faculdade de Medicina da Universidade de Sao Paulo Sao Paulo Brazil; ^12^ Servico de Controle de Infeccao Hospitalar, Instituto Central do Hospital das Clinicas, HCFMUSP, Faculdade de Medicina, FMUSP Universidade de Sao Paulo Sao Paulo Brazil

**Keywords:** *Aspergillus fumigatus*, *aspergillus* typing, clinical samples, environmental samples, short tandem repeats

## Abstract

**Background:**

Typing *Aspergillu*s species is crucial for understanding the sources of infection in hospital environments.

**Objectives:**

This study analysed clinical and air samples as well as their relationship with the clinical forms of aspergillosis. Additionally, we examined the usefulness of the Short Tandem Repeats (STR) technique with two highly discriminatory markers for analysing the *Aspergillus fumigatus* ( *A. fumigatus* ) profile.

**Patients/Methods:**

Seventy‐five air samples (September 2013–July 2014) and 116 clinical samples (2009–2014) were collected in a university hospital. Seventy‐two samples were typed by STR with two markers, MC3 and MC5.

**Results:**

Of the 75 air samples collected, 10 were positive in the Bone Marrow Transplant unit, a ventilated unit with HEPA filters as were 18 in the Haematology ward, a naturally ventilated unit. Of the 116 clinical samples of *Aspergillus* spp., 95 were identified as *A. fumigatus* . High diversity was found, with 42 genotypes in 67 clinical samples and four in five environmental samples. Most isolates were collected during the demolition and renovation of the Emergency unit in the Hospital from 2013 to 2014. Genotype 1 was found in several units during different years. Despite the heterogeneity, identical genotypes were observed three times at short intervals in the same or different wards. Some of these identical genotypes were confirmed as possible clones by genome sequencing while others' genotyping matches failed to be confirmed.

**Conclusion:**

Despite the diversity of clinical and environmental samples, useful correlations can be established in invasive aspergillosis surveillance programs by using this simple STR method as a preliminary step.

## 1 Introduction

Invasive fungal infections are associated with high morbidity and mortality rates among an increasing number of immunocompromised patients [1, 2]. *Aspergillus fumigatus* ( *A. fumigatus* ) is the most commonly recorded cause of infection in these patients, highlighting the importance of the hospital environment and the growing issue of azole resistance [3].

Diagnosis of fungal disease in severe cases is based on clinical signs and symptoms, culture, histopathology, and detection of fungal components such as galactomannan and (1,3)‐β‐D‐glucan, as well as PCR, which has recently been incorporated into diagnostics [4]. To reduce lethality caused by *Aspergillus*, early diagnosis using real‐time PCR associated with microbiological diagnosis has also been proposed [5].

Fungal identification and the usefulness of molecular methods that can provide details about sources of infection and epidemic outbreaks are valuable. Molecular typing was developed to replace phenotypic methods, which are less reliable and discriminatory [6]. Among them, the Short Tandem Repeats (STR) or a variable number of tandem repeats have shown high discriminatory power, as reported with nine markers divided into three multicolor multiplex PCRs [7, 8].

Using a set of eight pairs of primers in a single multiplex reaction to analyse 116 clinical isolates of *A. fumigatus*, a discriminatory power of 0.9997 (standard deviation of 0.0017) was demonstrated between presumably unrelated isolates [9]. The genotypic variation rate was 99.6% between groups of strains from three different health centers. Additionally, MC3 and MC5 were the most discriminating markers with 31 and 21 different alleles, respectively, followed by MC6a and MC6b with 17 alleles, MC 8 with 18 alleles, MC7 with 11 alleles, and MC1 and MC2 with 14 alleles [9].

Using the STR multiplex, high diversity was found in the environmental air and water samples collected in clinical units in 2010 [10] (160 different genotypes out of 250 *A. fumigatus* isolates). According to the authors, MC3 and MC5 markers were the most discriminatory at detecting microvariation between samples compared to the other markers [10].

Comparative analysis of surveillance of clinical respiratory and environmental samples using “Illumina” next‐generation sequencing, and short tandem repeats (STRs) revealed a lack of similarity between the strains (high diversity), which can be explained by the accumulation of *Aspergillus* spores in the hospital environment [11]. Applying STR genotyping in clinical settings enables us to understand how indoor fungi spread, assess infection risk, and implement measures to mitigate risk factors and control *Aspergillus* infections, particularly *A. fumigatus* .

This study analysed clinical and air samples collected from 2009 to 2011 and from 2013 to 2014 in order to establish links between environmental findings and invasive aspergillosis as well as other clinical manifestations of aspergillosis. From January 2013 to July 2014, the Emergency area of the Central Hospital building was renovated. This renovation was associated with an increased number of cases of invasive and non‐invasive aspergillosis. *A. fumigatus* was the most frequent isolate, and was genotyped by STR evaluation of two highly polymorphic markers. These markers could discriminate between *A. fumigatus* isolates present in the air and in clinical samples from patients admitted to a University Hospital.

## 2 Patients, Material and Methods

### 2.1 *Aspergillus* app. Isolates From Clinical Samples

From 2009 to 2011 and from 2013 to 2014, 116 *Aspergillus* spp. isolates from bronchoalveolar lavage, sputum, tracheal secretions, nasal secretions, surgical secretions, ascitic fluid, and biopsies from 107 patients were identified at the Laboratory of Medical Research in Microbiology—Division of the Central Laboratory—LIM 03 of the HC‐FMUSP. The patients were admitted to the Bone Marrow Transplant/Haematology (BMT‐Hemato), Pulmonology (Pulmo), Infectious Diseases, Kidney and Liver Transplantation wards of the Central Institute as well as the Children's, and Cancer Institutes of the Hospital das Clinicas da Faculdade de Medicina of the University of Sao Paulo (HC‐FMUSP). Repeated samples were collected from seven patients at intervals ranging from 1 day to 6 months. The laboratory is accredited by the American College of Pathology and adheres to all guidelines for performing fungal cultures. Samples were processed according to Clinical & Laboratory Standards Institute (CLSI) guidelines [12] and subcultured on Mycosel agar and Sabouraud dextrose agar with antibiotics (vancomycin 6 μg/mL and ciprofloxacin 21 μg/mL). The cultures were then incubated in aerobic conditions at 28°C (±2°C). During the incubation period, when macromorphology suggested growth of *Aspergillus* spp., a Scotch tape slide was performed. If *Aspergillus* section *Fumigati* species were confirmed by micromorphology, the result was validated. In cases of slow or atypical sporulation, a subculture on potato dextrose agar and Sabouraud dextrose agar was incubated in aerobiosis at 28°C (±2°C) for 7 days plus a slide culture was realized for *Aspergillus* section *Fumigati* identification. If *A. fumigatus* was present, the diagnosis was made but the culture continued to exclude other fungi.

*A. fumigatus* was identified by microbiological methods in 95 isolates and further confirmed by molecular methods, as described in Section *Aspergillus* species identification.

### 2.2 Classification of Aspergillosis

Clinical cases of *Aspergillus* spp. were classified retrospectively as invasive pulmonary aspergillosis (IPA, probable and proven, *n* = 25) and chronic pulmonary aspergillosis (CPA, *n* = 14), according to the European Organisation for Research and Treatment of Cancer/Mycoses Study Group criteria (EORTC/MSG) [4, 5, 13]. Subcutaneous surgical wound aspergillosis (SA) was also observed in two cases and was grouped with the 14 cases of chronic pulmonary (CPA) as CPSA (chronic pulmonary + subcutaneous aspergillosis, *n* = 16). Colonised patients (CO *n* = 66) were also reported. The total number of clinical isolates of *Aspergillus* spp. was, respectively, 29 from IPA cases, 16 from CPA, two from subcutaneous aspergillosis, and 69 from CO, due to an additional nine samples collected from seven of these patients.

### 2.3 Environmental Samples

From September 2013 to July 2014, 75 air samples were collected: 29 in the Bone Marrow Transplantation (BMT) Unit, which was ventilated with HEPA filters (in patient rooms and corridor) and 46 samples in the naturally ventilated areas of the Haematology ward (in patient rooms, corridors, and administrative room), which were close to the previous ward. This procedure was repeated three to four times in five different filtered and non‐filtered environments of Bone Marrow Transplantation or Haematology at intervals ranging from 1–4 to 1–10 months, and during each season of the year. Samples were collected for 10 min, about 1 m off the ground, in the morning, always in the same place as the previous sampling.

The RCS Microbial Air Sampling System 2024 Merck KGaA, Darmstadt, Germany uses the proven Reuter Centrifugal Impaction (RCS) principle, capable of gently and effectively collecting airborne microorganisms at low impaction speeds. There are seven preset sampling volumes and three user‐defined volumes ranging from one to 2000 L at a speed of 100 L/min. It was kindly provided by Laboratorio de Investigação Medica em Bacteriologia do HC‐FMUSP (LIM 54). From these 75 collected air samples, 28 *Aspergillus* spp. isolates were obtained.

### 2.4 Aspergillus Species Identification

DNA was extracted from clinical and air isolates according to the QIamp DNA tissue kit recommendations (Qiagen), which included an initial enzymatic lysis step with a buffer containing β‐mercaptoethanol and lyticase, followed by a mechanical lysis step containing glass beads.

For species identification, all samples were processed by PCR for β‐tubulin, Rodlet A, ITS1/4, and calmodulin genes at the Laboratorio de Investigacao Medica em Imunologia do HC‐FMUSP (LIM 48). The β‐tubulin gene segment was amplified using bT2a and bT2b primers [14], Rodlet A primers [15], and calmodulin primers [16, 17], in case of discordance between gene fragments.

These genes were sequenced and the fragments were analyzed separately. The results were first checked and compared with reference strains from GenBank (www.ncbi.nlm.nih.gov/genbank/), BLAST (www.ncbi.nlm.nih.gov/blast). Then, the results were aligned using the ClustalW (http://www.genome.jp/tools/clustalw/) and MUSCLE (www.ebi.ac.uk/Tools/msa/muscle) programs. Phylogenetic analyses were performed using the MEGA 6 program (http://www.megasoftware.net/mega.php).

Ninety‐five isolates were identified as *A. fumigatus* . Short Tandem Repeat (STR) analysis was performed on 72 of the *A. fumigatus* isolates (67 clinical isolates from 18 IPA, 10 CPA + SA aspergillosis, 39 CO patients, and five environmental isolates). Sixty of these isolates were obtained from the clinical samples of 60 patients. An additional seven isolates were collected from five of these patients in a minimum interval of one‐eight days and a maximum interval of 57 days. According to Araujo et al. 2009, two markers with high discriminatory power, MC3 and MC5, were used [9]. The samples were subjected to capillary electrophoresis in the ABI310 Genetic Analyser sequencer (Applied Biosystems) using the POP‐4 polymer (Applied Biosystems). As controls, six strains of *A. fumigatus* (ATCC 46645, L02, J21, F68, L25, G01) were kindly provided by i3S, at University of Porto, Portugal.

We determined the relative size of the fragments using the GeneMapper ID v3.2 (Applied Biosystems) software, which calculates fragment size in base pairs (bp) of the amplified products using known peak sizes from the molecular size standard as a reference.

### 2.5 Whole Genome Sequencing

For library preparation, 100 ng of genomic DNA was used, following the previously described extraction protocol. The template preparation was performed by the Ion Chef System (Thermo Fisher Scientific, MA, USA), using the Ion 510 & Ion 520 & Ion 530 Kit—Chef (Thermo Fisher Scientific, MA, USA). The Ion Chef system automates template preparation, including emulsion PCR (emPCR) and Ion Sphere particle enrichment (ISPs). After this step, the samples were loaded onto the Ion 530 chips. The sequencing was conducted in the Ion S5 System (Thermo Fisher Scientific, MA, USA), and the race control, the base call, and the initial quality analyses were performed by the Torrent Suite software, integrated into the Ion S5 system.

The raw sequencing reads in FASTQ format were quality‐trimmed using Trimmomatic (v0.39) to remove low‐quality bases and adapter sequences. Quality control was assessed before and after trimming using FastQC.

### 2.6 De Novo Genome Assembly and Polishing

The trimmed reads were assembled *de novo* using SPAdes (v3.15.5) with the default parameters. The resulting assemblies were polished with Pilon (v1.24) to correct potential base‐calling errors and indels. Assembly quality was evaluated using QUAST (v5.2.0).

*In silico* MLST analysis was performed using the *Aspergillus fumigatus* scheme available in the PubMLST database (https://pubmlst.org/bigsdb?db=pubmlst_afumigatus_seqdef). For phylogenetic reconstruction, contigs were aligned to the reference genome Af293 using RealPhy (v1.12 https://realphy.unibas.ch/realphy/) [18], and SeaView (v. 5) [19], to construct a maximum likelihood tree based on the core genome alignment with a bootstrap of 100 replicates.

### 2.7 Principal Component Analysis (PCA) of SNP Profiles

For SNP‐based population structure analysis, trimmed reads were mapped to the reference genome Af293 (GCF_000002655.1) using BWA MEM (v 0.7.18). The alignment files in BAM format were processed using FreeBayes (v1.3.6) for variant calling. SNPs were filtered by quality (Q 30) and coverage (10×) and then used to build a genotype matrix. This matrix was subjected to Principal Component Analysis (PCA). Visualisation of PCA results was conducted in RStudio 2024.09.1 using the ggplot2 and plotly packages.

### 2.8 Data and Statistical Analyses

Demographic and clinical data were collected from the medical records, and the results from the molecular tests were organised in Excel tables. A preliminary analysis was performed using the chi‐square test and Fisher's exact test for categorical or nominal independent variables. We tested the normality of continuous data using the Kolmogorov–Smirnov test. If the data showed a normal distribution, the *T*‐test for independent samples was performed. SPSS (version 26.0, IBM, New York) was used for this analysis. The dendrogram was constructed using Euclidean distance and the UPGMA clustering method with BioNumerics (v. 7.6.2, Applied Maths NV, Sint‐Martens‐Latem, Belgium). Isolates were clustered based on 97% similarity and labelled A through J. Statistical analysis for pairwise linkage disequilibrium was performed by an exact test using a Markov chain (chain length of 10,000 and dememorization of 1000), and the statistical software package Arlequin 3.1 (http://cmpg.unibe.ch/software/arlequin3/). Genotype diversity (discriminatory power) analysis of molecular variance (AMOVA) and fixation indices were also tested using Arlequin software.

### 2.9 Ethics and Research Committee

This protocol was approved by the Ethics Committee for Analysis of Research Projects—CAPPesq of the Clinical Board of the Hospital das Clinicas of the Faculty of Medicine of the University of Sao Paulo as the research protocol number 0156/08. Patient data were obtained retrospectively, and secrecy and confidentiality were ensured.

## 3 Results

From 2009 to 2011 and from 2013 to 2014, 13 species of *Aspergillus* spp. were identified among the fungal isolates obtained from clinical and environmental samples (Table 1). Twenty‐eight environmental isolates were obtained from 29 samples collected in filtered rooms and corridors (10 isolates total) and 46 samples collected in non‐filtered environments (18 isolates total) of the Bone Marrow Transplantation and Haematology wards (BMT‐Hemato). Sixteen isolates were identified from non‐filtered rooms. More frequently, the isolates were *A. tamarii*, *A. flavus* and *A. fumigatus* (Table 1). Of the 12 isolates from filtered rooms, *A. parasiticus* was the most common, as seen in Table 1. A total of 95 clinical isolates and five environmental isolates (two from HEPA‐filtered rooms and three from unfiltered environments) were identified as *A. fumigatus* .

**TABLE 1:** *Aspergillus* spp. from 116 clinical isolates from 107 patient samples and 28 air samples.

*Aspergillus* spp.	Clinical isolates	Air isolates			Total
		Filter	Non‐filtered	Total	
*A. fumigatus*	95	2	3	5	10
*A. flavus*	6	2	4	6	12
*A. clavatus*	3	0	0	0	3
*A. awamori*	1	0	0	0	1
*A. terreus*	1	0	0	0	1
*A. niger*	9	0	2	2	11
*A. amoenus*	0	1	1	2	2
*A. japonicus*	0	0	1	1	1
*A. ochraceus*	0	1	0	1	1
*A. parasiticus*	0	3	0	3	3
*A. sydowii*	0	1	1	2	2
*A. tamarii*	0	1	4	5	5
*A. welwitschiae*	1	1	0	1	2
***Total***	**116**	**12**	**16**	**28**	

Both *A. fumigatus* and other species were mainly isolated from CO patients, followed by IPA patients (Table 2). IPA was associated with *A. fumigatus* in 20 patients, *A. flavus*, in four patients and *A. niger* in one patient. The most common comorbidities were cystic fibrosis and Haematopoietic Stem Cell Transplantation (Table 2). *Aspergillus fumigatus* and non‐*fumigat*us isolates were more frequent in summer and autumn (*p* = 0.044, Table 2).

**TABLE 2:** Distribution of 107 patients with *A. fumigatus* and other *Aspergillus* spp. according to clinical, biological, and sociodemographic variables.

Variable	*A. fumigatus*	Other species	Total patients	*p*
	80.4% (*n* = 86)	19.6% (*n* = 21)	100% (*n* = 107)	
Age (years)				
Mean ± SD	43.53 ± 19.05	54.43 ± 17.34	46.08 ± 18.9	**†0.019**
Lower‐Upper	1–86	18–83	1–86	
Sex				
Female % (*n*)	51.2 (44)	66.7 (14)	54.2 (58)	‡0.201
Clinical Forms % (*n*)				
CO	60.5 (52)	66.7 (14)	61.7 (66)	§0.883
IPA	23.3 (20)	23.8 (5)	23.4 (25)	
CPSA (CPA + SA)	16.3 (14)	9.5 (2)	15.0 (16)	
Comorbidities % (*n*)				
Cystic fibrosis/bronchiectasis	29.7 (22)	47.4 (9)	33.3 (31)	§0.359
Transplantation	27.0 (20)	21.1 (4)	25.8 (24)	
Tuberculosis	16.2 (12)	10.5 (2)	15.1 (14)	
Leukaemia, Lymphoma, Myeloma	9.5 (7)	15.8 (3)	10.8 (10)	
Chronic respiratory diseases	4.1 (3)	5.3 (1)	4.3 (4)	
Others	13.5 (10)	0 (0)	10.8 (10)	
Missing	13.9 (12)	0.1 (2)	13.1 (14)	
Origin clinics % (*n*)				
Pulmonology	67.4 (58)	71.4 (15)	68.2 (73)	§0.301
BMT‐Hemato	15.1 (13)	23.8 (5)	16.8 (18)	
Others	17.4 (15)	4.8 (1)	15.0 (16)	
Clinical samples % (*n*)				
Bronchoalveolar Lavage	43.0 (37)	52.4 (11)	44.9 (48)	§0.129
Sputum/tracheal secretion	39.5 (34)	28.6 (6)	37.4 (40)	
Biopsy	7.0 (6)	19.0 (4)	9.3 (10)	
Others	10.5 (9)	0 (0)	8.4 (9)	
Year				
2009	9.3 (8)	4.8 (1)	8.4 (9)	§0.460
2010	12.8 (11)	9.5 (2)	12.1 (13)	
2011	3.5 (3)	4.8 (1)	3.7 (4)	
2013	51.2 (44)	38.1 (8)	48.6 (52)	
2014	23.3 (20)	42.9 (9)	27.1 (29)	
Seasons				
Summer	24.4 (21)	52.4 (11)	29.9 (32)	*§**0.044**
Autumn	31.4 (27)	33.3 (7)	31.8 (34)	
Winter	25.6 (22)	9.5 (2)	22.4 (24)	
Spring	18.6 (16)	4.8 (1)	15.9 (17)	

*Note:* CO – Colonised; Transplantation: *n* = 20 Haematopoietic Stem Cell Transplantation, 2 kidney, 2 liver; IA– Invasive Pulmonary Aspergillosis: CPSA—Chronic Pulmonary (*n* = 14) + Subcutaneous Aspergillosis (*n* = 2); BMT–Hemato = Bone Marrow Transplantation‐Haematology. Clinics – Others: Infectology, Dermatology, Neurology, Paediatrics, Cancer Institute. Samples – Others: surgical and nasal secretions, ascitic fluid. Statistical analyses: *p = 0.044, the difference in the summer between *A. fumigatus* and other species. Statistics: †T test; ‡Chi‐square test; §Fisher's Exact test. *p* ≤ 0.05 was significant for all tests.

Higher rainfall and temperatures occur in the summer while lower rainfall and temperatures occur in the winter.

As seen in Table 3, most of the isolates were collected in the autumn and summer, and in both semesters of 2013 and the first semester of 2014. From January 2013 to July 2014, the hospital's emergency unit, located in the same building as most of the wards, underwent a major renovation. Sixty percent of patients with invasive aspergillosis were reported in 2013, and the most common associated comorbidities were transplantation and haematological neoplasms. Cystic fibrosis/bronchiectasis was associated with colonised cases. In addition to the BMT‐Hemato wards, invasive aspergillosis samples were also collected in the Pulmonology ward, whereas specimens from patients with chronic aspergillosis were collected in the Pulmonology and other wards (Table 3).

**TABLE 3:** Distribution of 107 patients according to the presence of invasive pulmonary aspergillosis, comorbidities, and sociodemographic variables.

Variable	IPA			*p*
	NO (*n* = 82)	YES (*n* = 25)	Total (*n* = 107)	
Age (years)				
Mean ± SD	**45.17 ± 20.12**	**47.32 ± 15.80**	**42.32 ± 20.19**	¶**0.029**
Lower‐Upper	1–86	12–73	1–86	
Sex	**(*n* = 47)**	**(*n* = 11)**	**(*n* = 58)**	
Female % (*n*)	43.9	10.3	54.2	‡0.173
Clinical Forms % (*n*)				
CO	80.5 (66)	0 (0)	61.7 (66)	§**0.000**
IPA	0 (0)	100.0 (25)	23.4 (25)	
CPSA (CPA + SA)	19.5 (16)	0 (0)	15.0 (16)	
Comorbidities % (*n*)	**75**	18	93	
Cystic fibrosis, bronquiectasis	38.7 (29)	11.1 (2)	33.3 (31)	§**0.005**
Transplantation*	24.0 (18)	33.3 (6)	25.8 (24)	
Tuberculosis	17.3 (13)	5.6 (1)	15.1 (14)	
Leukaemia/Lymphoma/Multiple myeloma	5.3 (4)	33.3 (6)	10.8 (10)	
Chronic respiratory diseases	5.3 (4)	0 (0)	4.3 (4)	
Others	9.3 (7)	16.7 (3)	10.8 (10)	
Origin clinics % (*n*)				
Pulmonology	78.0 (64)	36.0 (9)	68.2 (73)	§**0.000**
BMT‐Hemato	3.7 (3)	60.0 (15)	16.8 (18)	
Others	18.3 (15)	4.0 (1)	15.0 (16)	
Clinical samples % (*n*)				
Bronchoalveolar lavage	41.5 (34)	56.0 (14)	44.9 (48)	§**0.023**
Sputum/tracheal secretion	43.9 (36)	16.0 (4)	37.4 (40)	
Biopsy	6.1 (5)	20.0 (5)	9.3 (10)	
Others	8.5 (7)	8.0 (2)	8.4 (9)	
Year				
2009	7.3 (6)	12.0 (3)	8.4 (9)	§0.205
2010	10.9 (9)	16.0 (4)	12.1 (13)	
2011	4.9 (4)	0 (0)	3.7 (4)	
2013	45.1 (37)	60.0 (15)	48.6 (52)	
2014	31.7 (26)	12.0 (3)	27.1 (29)	
Seasons				
Summer	32.9 (27)	20.0 (5)	29.9 (32)	§0.492
Autumn	31.7 (26)	32.0 (8)	31.8 (34)	
Winter	19.5 (16)	32.0 (8)	22.4 (24)	
Spring	15.9 (13)	16.0 (4)	15.9 (17)	

*Note:* CO – Colonised; IPA– Invasive Pulmonary Aspergillosis; CPSA = CPA – Chronic Pulmonary Aspergillosis (*n* = 14), Subcutaneous Aspergillosis (*n* = 2); BMT–Hemato—Bone Marrow Transplantation‐Haematology. Transplantation—*n* = 20 Haematopoietic Stem Cell Transplantation, 2 kidney, 2 liver; Others: Infectology, Dermatology, Neurology, Paediatrics, Cancer Institute. Six patients collected repeated samples, the total number of clinical samples = 116. Missing values for comorbidities (NO IPA = 7, IPA = 7, Total = 14). Statistics: ‡Chi‐square test; §Fisher's Exact test; ¶Kolmogorov–Smirnov test. *p* ≤ 0.05 was significant for all tests.

Samples from patients with IPA were collected in the BMT ward, with HEPA filters, but more frequently in the unfiltered Pulmonology and Haematology wards than in other wards, especially during the renovation period. Some patients were previously admitted to the Emergency unit and had positive specimens collected in this unfiltered area.

A subset of 67 *A. fumigatus* isolates (71%) from 60 patients was used for STR genotyping. The distribution of patients according to sociodemographic and clinical variables is shown in Table S1. These 60 patients' characteristics are similar to those of the full set of patients used in the full study (see Table 3).

Table 4 shows the distribution of 42 genotypes (38 from clinical samples and four from environmental samples) and the number of isolates within each genotype, according to the source (air or clinical), clinical form, ward, or year of collection. Genotype 1 was the most prevalent among clinical isolates, and no differences were observed between patients with IPA and those with other clinical diseases.

**TABLE 4:** Distribution of genotypes among the set of 72 clinical and environmental isolates analysed.

Geno types	Total	Samples (*n* = 72)		Clinical forms (*n* = 67)			Wards % (*n* = 72)			Year % (*n* = 72)			
		Clinical	Environmental	IPA	CO	CPSA	BMT/Hem	Pulmo	Others	2009	2010/2011	2013	2014
1	11	9	2	2	6	11	4	6	1	2	0	6	3
2	1	0	1	0	0	0	1	0	0	0	0	0	1
3	2	2	0	2	0	0	2	0	0	0	2	0	0
4	3	3	0	2	0	1	1	1	1	0	0	3	0
5	1	1	0	0	1	0	0	1	0	0	1	0	0
6	1	1	0	0	1	0	0	0	1	0	0	0	1
7	1	1	0	1	0	0	0	0	1	1	0	0	0
8	1	1	0	1	0	0	1	0	0	0	0	1	0
9	1	1	0	0	1	0	0	0	1	1	0	0	0
10	1	1	0	0	0	1	0	1	0	0	0	0	1
11	1	1	0	0	1	0	0	0	1	0	0	1	0
12	1	1	0	0	1	0	0	1	0	0	1	0	0
13	1	1	0	0	1	0	0	1	0	0	0	1	0
14	5	5	0	2	3	0	3	1	1	4	0	1	0
15	1	1	0	0	1	0	1	0	0	0	0	0	1
16	2	2	0	0	1	1	0	1	1	0	0	1	1
17	1	1	0	0	1	0	0	1	0	0	1	0	0
18	1	1	0	0	1	0	0	0	1	0	0	1	0
19	3	3	0	0	2	1	1	1	1	1	2	0	0
20	1	1	0	1	0	0	1	0	0	0	1	0	0
21	3	3	0	1	2	0	0	2	1	0	0	2	1
22	1	1	0	1	0	0	1	0	0	0	0	1	0
23	1	1	0	0	0	1	0	0	1	0	1	0	0
24	1	1	0	0	1	0	0	1	0	0	0	1	0
25	1	1	0	0	1	0	0	1	0	0	0	1	0
26	2	1	1	0	0	1	1	1	0	0	0	1	1
27	1	1	0	1	0	0	0	1	0	0	1	0	0
28	4	4	0	0	3	1	0	3	1	0	2	2	0
29	2	2	0	0	2	0	0	2	0	0	0	0	2
30	1	1	0	0	0	1	0	0	1	1	0	0	0
31	1	1	0	1	0	0	1	0	0	0	0	0	1
32	2	1	1	0	1	0	1	1	0	0	0	2	0
33	1	1	0	0	1	0	0	1	0	0	0	1	0
34	1	1	0	0	1	0	0	1	0	0	0	0	1
35	2	2	0	0	0	2	0	1	1	0	0	2	0
36	1	1	0	0	1	0	0	1	0	0	0	1	0
37	1	1	0	1	0	0	1	0	0	0	1	0	0
38	2	2	0	0	2	0	0	2	0	1	0	1	0
39	1	1	0	1	0	0	0	1	0	0	0	1	0
40	1	1	0	0	1	0	0	1	0	0	0	1	0
41	1	1	0	0	1	0	0	1	0	0	0	1	0
42	1	1	0	1	0	0	0	1	0	0	0	0	1
		**67**	**5**	**18**	**38**	**11**	**20**	**37**	**15**	**11**	**13**	**33**	**15**

*Note:* IPA‐ invasive pulmonary aspergillosis; CO – colonised; CPSA – chronic pulmonary + subcutaneous aspergillosis; BMT/Hem – Bone Marrow Transplantation/Haematology; Pulmo‐ Pulmonology; Others—Infectology, Dermatology, Neurology, Paediatrics, Cancer Institute.

**FIGURE 1:**
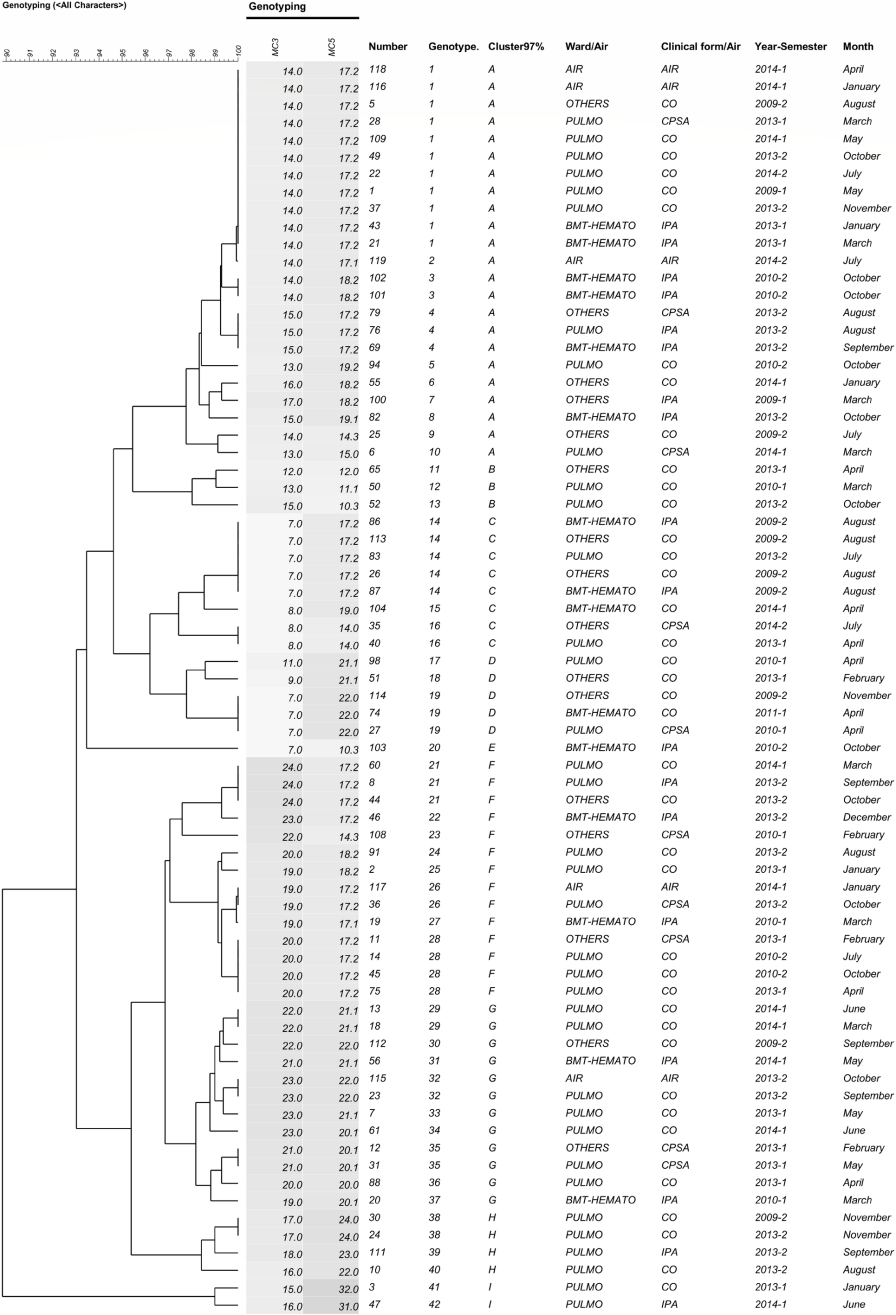
Dendrogram generated from STR comparison of 72 clinical and environmental *Aspergillus fumigatus* isolates genotyped by MC3 and MC5 markers. The dendrogram was constructed using Euclidean distance and the UPGMA clustering method. Number = sample number; BMT‐HEMATO: Bone Marrow Transplantation‐Hematology; PULMO – Pulmonology; CO – Colonisation (*n* = 39); CPSA – Chronic pulmonary (*n* = 8) + subcutaneous aspergillosis (*n* = 2); IPA – invasive pulmonary aspergillosis (*n* = 18); AIR – environmental samples from Bone Marrow Transplantation‐Hematology ward (*n* = 5).

Regarding the semester, similar genotypes were shown for environmental and clinical samples in semesters 2013–1, 2013–2, and 2014–1. Only one of the five air isolates did not match clinical samples but had a variation in one marker (Figure 1).

Figure 1 shows the dendrogram considering the genotyping results for the MC3 and MC5 markers, sample type (clinical or environmental), ward of admission/sampling, semester, and year of collection. After analyzing both microsatellite loci, 42 genotypes were found among the 72 isolates (38 genotypes in 67 clinical and four in 5 environmental isolates).

**FIGURE 2:**
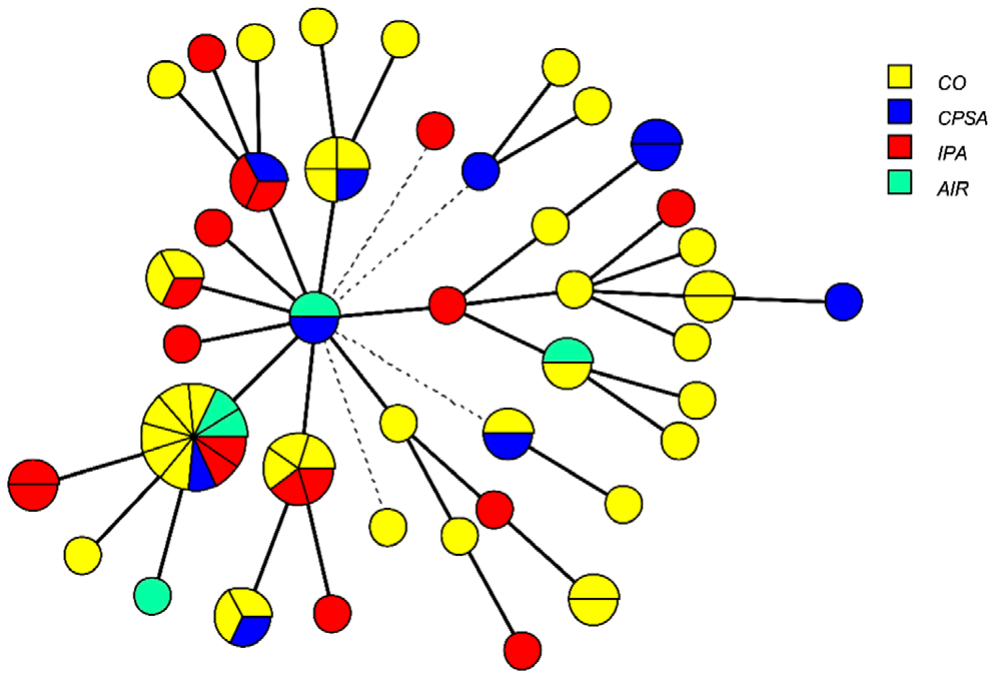
Minimum spanning tree showing the relationship between clinical and environmental *Aspergillus fumigatus* isolates genotyped by MC3 and MC5 markers and clinical forms. Seventy‐two isolates were distributed in 42 genotypes represented by circles. Their sizes represent the number of isolates for each genotype, colored by samples from different clinical forms, patients, and environmental samples. Solid lines indicate variation in one marker, while dashed lines indicate variation in both markers. CO – Colonisation (*n* = 37); CPSA – Chronic pulmonary (*n* = 14) + subcutaneous aspergillosis (*n* = 2); IPA – invasive pulmonary aspergillosis (*n*= 14); AIR – environmental samples from Bone Marrow Transplantation‐Hematology ward (*n* = 5).

“Minimum spanning trees” showing the relationship between clinical and environmental *Aspergillus fumigatus* isolates genotyped by MC3 and MC5 markers are presented, in Figure 2, according to the clinical form (IPA, CPSA, and CO), and in Figures S1 and S2, according to the wards and semesters of the year. Some samples from colonised patients, invasive aspergillosis patients, and chronic aspergillosis patients and air isolates had the same genotype (Figure 2).

The number of repeats in MC3 and MC5 markers for each genotype based on raw sequencing data is shown in Table S2 and electrophoretic peaks for the MC3 and MC5 markers tested on two isolates are shown in Figure S3.

Genotype 1 was the most commonly observed genotype, appearing in clinical isolates in 2009, 2013 and 2014. It was also found in patients admitted to different wards, including the BMT‐Haematology ward and other wards within the same hospital in 2013.

Additionally, this genotype was isolated from environmental samples collected in 2014.

Furthermore, Genotype 1 was detected in air samples collected in the BMT‐Haematology ward, as well as in clinical samples from Pulmonology, BMT‐Haematology, and other wards (see Figure S1). The Pulmonology ward contributed the most clinical samples due to colonisation (see Figures 2 and S1). Genotype 1 (see Figures 1 and S2) was isolated in clinical samples from 2009, 2013 and 2014, as well as in air samples from 2014 (see Figure S2). A second genotype (number 32) was prevalent in both air and clinical samples in 2013. Additionally, a third genotype (number 26) was shared between a clinical sample collected in 2013 and an air sample collected 3 months later in 2014.

Despite multiple genotypes being present among patients admitted to the same ward, we occasionally identified identical genotypes in patients with IPA, CPSA and CO. In the winter of 2009, genotype 14 was isolated within 2 days in three patients admitted to two different wards. Two of the patients had CO and stayed in different unfiltered rooms on the same floor. The third patient had IPA and stayed in a filtered room but circulated in the same area for medical procedures. Genotype 14 was also identified in winter of 2013 in another ward. In 2013, genotype 1 was observed in three CO patients staying in the Pulmonology ward (which lacked an air filtration system) within 43 days of each other. Also in 2013, genotype 4 was observed in three patients (two with IPA and one with CPSA) who were admitted within 50 days of each other and stayed in one filtered and two non‐filtered rooms.

We observed identical genotypes in clinical samples collected from the same patient at one‐ and five‐day intervals (samples 86–87 and 101–102, respectively) within the same ward. However, different genotypes were observed in all other cases of repeated samples collected within 43 days (Figure 1). Five repeated samples showed a difference in one marker.

In terms of genetic diversity, the Simpson index was 0.91 for marker MC3 and 0.82 for marker MC5. For both markers together, the Simpson index was 0.97.

The AMOVA test revealed that there were no significant differences among groups of strains from different origins (clinical versus environmental), collection years (2009, 2010, 2013 and 2014), or clinical forms (CO, IPA and CPSA). More than 97.5% of the genotypic variation was intrapopulational for all three comparisons. Fixation indices between populations were not significant at the single‐locus level for any of the comparisons among the groups of strains from different origins, collection years, or clinical forms (data not shown).

A small set of six isolates (four classified with genotype 1 and two with genotype 32) was used for the complete genome sequence. The results showed less than 3900 SNPs of difference between these genomes, the isolate 118 (with genotype 1) being the most different of this set. The isolates 21 and 116 (both with genotype 1) were the most similar, showing a difference of 1300 SNPs in their genomes; these isolates may be considered clones, as principal component analysis based on SNPs showed such similarity (Figure S3). When these six genomes were compared with a larger set of genomes of *A. fumigatus* obtained from NCBI, isolate 118 was the outlier and grouped with other genomes from the USA, while the remaining five genomes from this study (classified with genotypes 1 and 32) were in a different clade (Figure S4).

## 4 Discussion

This study describes several species other than *A. fumigatus* in wards with and without HEPA filters. These species are clinically important due to their lower sensitivity to antifungal agents. *A. parasiticus* , which is predominant in environments with filters, is less sensitive to amphotericin; *A. tamarii*, which is predominant in environments without filters, is azole‐resistant. *A. sydowii* is also azole‐resistant, as previously described [20, 21].

Given the severity of IPA, the need for early and accurate diagnosis is emphasized. While most IPA cases occurred in immunosuppressed haematological patients in Bone Marrow Transplantation and Haematology wards, over 30% of cases of IPA had comorbidities such as tuberculosis, paracoccidioidomycosis, HIV infection, cystic fibrosis, granulomatous disease and patients were hospitalized in wards such as Pulmonology and Neurology. Furthermore, over 20% of IPA cases were caused by *Aspergillus* species other than *fumigatus* (four by *A. flavus* and one by *A. niger* ), which have different sensitivities to antifungals. Notably, 60% of IPA cases occurred in 2013, likely due to increased spore circulation during the hospital's renovation period [22, 23].

Considering only two microsatellite markers, we found high variability of *A. fumigatus* . A total of 38 genotypes were observed among 60 patient isolates and four genotypes among five environmental isolates. These observations are consistent with previous reports using similar markers [10, 24, 25]. Such high genetic diversity in clinical and environmental samples suggests that clinical samples of *A. fumigatus* are unrelated, and that *A. fumigatus* spores accumulate within the hospital environment rather than originating from a single source responsible for fungal reproduction [11]. The presence of the same genotypes (1, 19, 28) in several wards supports this idea.

Previous studies have reported the persistence of specific genotypes in the environment or in patient samples from the same or different units, or even in another building over the years (2009–2014) [25, 26], confirming that colonisation by this fungus may have originated from a local source within the hospital environment [25], followed by indoor air exchange. Transferring patients between rooms with and without filtered air, as well as transferring them to different intensive care units (unfiltered areas), may have contributed to fungal colonisation during their stay. Additionally, the presence of areas under reconstruction or renovation close to wards housing our patients could explain the high number of fungi and genotypes present in air samples collected during the 2013–14 period. Increased levels of fungal spores, particularly *A. fumigatus* , are expected during periods of renovation and demolition in a hospital environment [22, 23]. Unfortunately, environmental isolates were not isolated or typed before 2013 in the BMT‐Haematology ward or the other wards included in this study.

The short intervals between the isolation of *A. fumigatus* clones with the same genotype from several patients in different wards may suggest a single source for all clones, followed by air colonisation, as previously described [9, 27].

In addition to the previously reported presence of similar genotypes isolated from the same patients within a short time frame [9], infections by multiple genotypes in repeated samples may result from patients being exposed to different fungal genotypes while moving around the hospital, especially during ward remodelling or construction work [23]. Furthermore, microvariation phenomena could occur [10]. The presence of the same genotype in patients exhibiting different clinical forms was frequently observed in our samples collected from different wards and over different periods (Figure 1). This may depend on the degree of host immunity and the presence of comorbidities that predispose individuals to invasive or chronic aspergillosis or colonisation (Table 3).

As shown, more than 97.5% of the genotypic variation is intrapopulation, independent of time (year of isolation, place, or origin of the strain). Analysis of genetic diversity showed no significant differences among groups of strains from different origins (clinical versus environmental), collection years (2009, 2010, 2013, and 2014), or clinical forms (CO, IPA and CPSA).

An interesting point raised by Guinea et al. [24] was the selection of improved, inexpensive, and rapid genotyping methods that provide unambiguous genotyping data. Our method appears reliable as demonstrated by its high variability in the clinical samples of 60 patients and its high discriminatory power (Simpson index = 0.97). Although we did not use eight or nine markers as previously proposed [7, 9], we selected two with the highest discriminatory power [10], as previously described successfully in *Candida* [28, 29, 30, 31]. One disadvantage of this method is that it requires expensive equipment and skilled personnel, which makes it costly. Nevertheless, it is feasible, and based on our results, these two highly discriminatory markers could be used as an initial, practical strategy for *A. fumigatus* typing, at least. An alternative is whole genome analysis, which has high discriminatory power and interlaboratory comparability [11], but it is much more expensive and requires highly specialised personnel. The analysis of a small set of genomes from isolates of this study confirmed the similarity of some isolates previously observed by the suggested microsatellite typing analysis (e.g., isolates 21 and 116) but failed to show such similarity among other isolates (e.g., isolates 23 and 115).

The strengths of this work are the association found between clinical and environmental genotypes during the renovation period in the hospital and the characterisation of the clinical epidemiological data of patients admitted to hospital wards. Additionally, this simplified method with only two markers identified high genetic variability in clinical and environmental isolates and revealed multiple matches between air and clinical samples. The study limitations include the low number of air samples with *A. fumigatus* obtained in 75 samples collected in the hospital, although this was expected in areas with air filtration systems. The comparison of larger collections of genomes of *A. fumigatus* collected from the same clinical environment can certainly clarify which epidemiological cases may benefit from microsatellite typing.

## 5 Recommendation

In addition to regular microbiological assessments of indoor hospital environments during renovations or constructions, regular environmental fungal sampling may help to prevent patient exposure to dust and elevated fungal concentrations [23]. Active surveillance of clinical wards is necessary to prevent colonisation and infection of immunosuppressed patients, as well as possible outbreaks. This plan includes systematically screening hospitalised patients and controlling air quality before, during, and after renovations or construction.

## 6 Conclusion

Despite the diversity of clinical and environmental samples, this study's findings suggest that useful correlations can be established during invasive aspergillosis surveillance programs using a simple STR typing method with polymorphic markers as a preliminary step.

## Author Contributions

**Claudia de Abreu Fonseca:** investigation, methodology, formal analysis, data curation, writing – review and editing, writing – original draft, software. **Ricardo Araujo:** writing – original draft, writing – review and editing, formal analysis, methodology, software. **Vivian Caso Coelho:** investigation, writing – review and editing, supervision. **Carlos Henrique Camargo:** software, formal analysis, writing – review and editing. **Marcello Mihailenko Chaves Magri:** investigation, methodology, writing – review and editing. **Adriana Lopes Motta:** investigation, methodology, writing – review and editing. **Marina Farrel Côrtes:** methodology, formal analysis, writing – review and editing, software. **Ana Carolina Mamana:** methodology, investigation, writing – review and editing. **Marjorie Vieira Batista:** methodology, investigation, writing – review and editing. **Daniel Valério da Silva Moreira:** methodology, investigation, writing – review and editing. **Ana Paula Croce:** methodology, investigation, writing – review and editing. **Mauro Cintra Giudice:** methodology, investigation. **André Nathan Costa:** investigation, writing – review and editing. **Sergio Eduardo Demarzo:** investigation, writing – review and editing. **Alexandra Gomes dos Santos:** investigation, writing – review and editing. **Thais Guimarães:** investigation, writing – review and editing, visualization. **Vera Lucia Teixeira de Freitas:** formal analysis, writing – review and editing, methodology, software. **Sílvia Figueiredo Costa:** investigation, writing – review and editing, formal analysis, supervision. **Maria Aparecida Shikanai Yasuda:** investigation, funding acquisition, validation, visualization, writing – review and editing, writing – original draft, project administration, supervision, conceptualization, formal analysis.

## Conflicts of Interest

The authors declare no conflicts of interest.

## Supporting information


**Figure S1:** Minimum spanning tree showing the relationship between clinical and environmental *Aspergillus fumigatus* isolates genotyped by MC3 and MC5 markers and the collection ward.

**Figure S2:** Minimum spanning tree showing the year of collection of clinical and environmental *Aspergillus fumigatus* isolates genotyped by MC3 and MC5 markers.

**Figure S3:** Electrophoretic peaks for MC3 and MC5 markers testing the strains 65 (A1, A2) and 112 (B1, B2) of *Aspergillus fumigatus* .

**Figure S4:** (A) Principal component analysis for the six genomes of this study using the SNPs between the genomes (PC1 represents the first principal component, PC2 the second principal component and PC3 the third principal component), and (B) Phylogenetic tree of *Aspergillus fumigatus* genomes available at NCBI and six genomes sequenced in this study (marked with *).

**Table S1:** Distribution of 60 patients according to socio‐demographic and clinical variables.

**Table S2:** Number of repeats in markers MC3 and MC5 for each genotype based on raw sequencing data.

## Data Availability

The molecular sequence data used in this study are available at GenBank (NCBI) under the following accession numbers:
SUB15668669 PX433666, PX433667, PX433668, PX433669, PX433670, PX433671, PX433672, PX433673, PX433674, PX433675, PX433676, PX433677, PX433678, PX433679, PX433680, PX433681, PX433682, PX433683, PX433684 PX438571, PX438572, PX4438573, PX4438574, PX438575, PX438576, PX438577, PX438578, PX438579, PX438580, PX438581, PX438582, PX438583, PX438584, PX438585, PX43586. Whole genome sequences are available under the following accession numbers: SAMN50834995, SAMN50834996, SAMN50834997, SAMN50834998, SAMN50834999 and SAMN50835000.
