# Supplementary figures and images for: Analysis of Aspergillus spp. Isolates According to Temporal–Spatial, Sociodemographic, and Clinical Variables—Microsatellite Typing of Clinical and Environmental Samples of Aspergillus fumigatus in a University Hospital in Sao Paulo, Brazil
Source: Mycoses. 2026 Jan 17;69(1):e70126. doi: 10.1111/myc.70126 (PMC12811795; doi:10.1111/myc.70126)

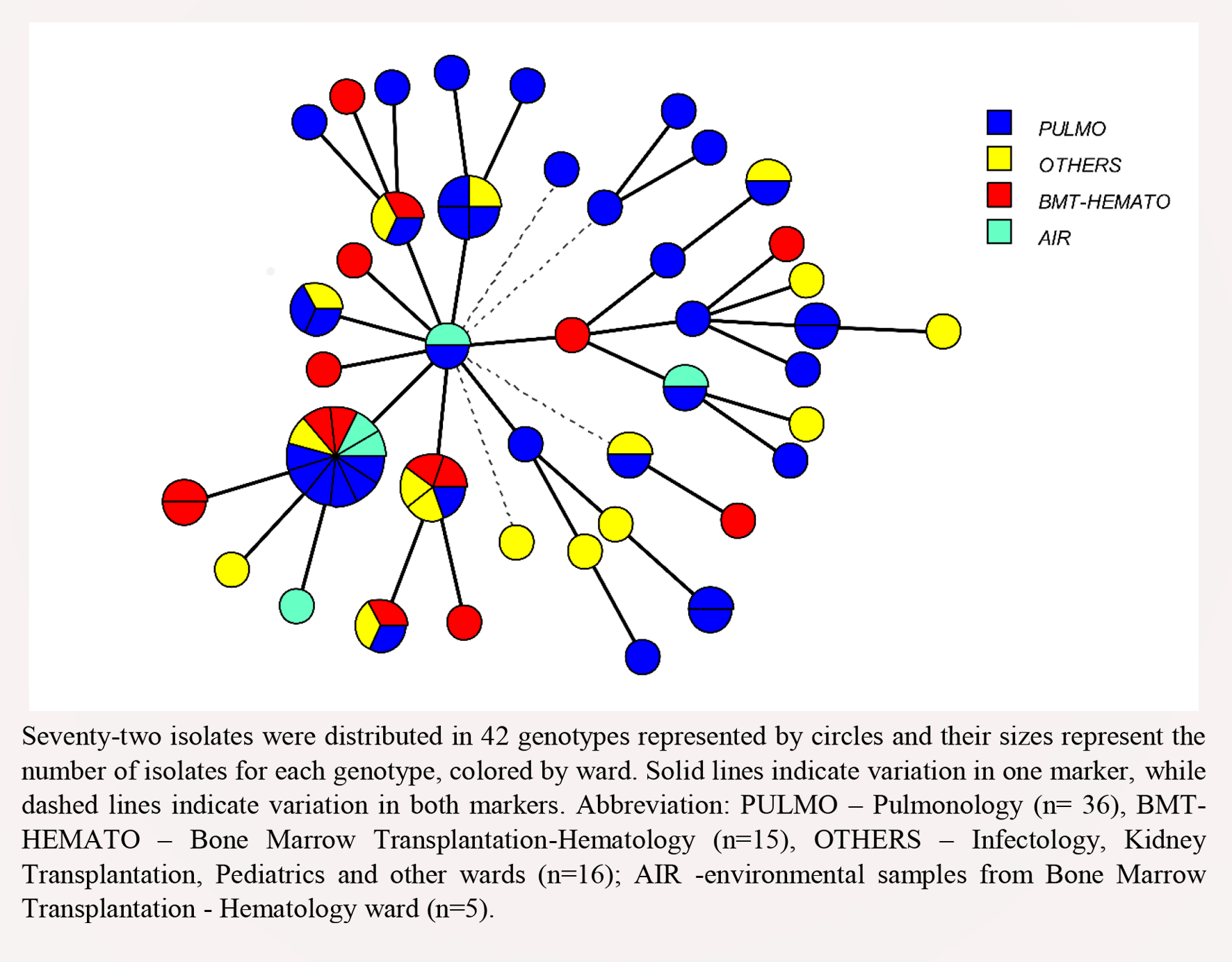

Supplement: Supplementary file 1 — Figure S1: Minimum spanning tree showing the relationship between clinical and environmental Aspergillus fumigatus isolates genotyped by MC3 and MC5 markers and the collection ward. [file MYC-69-e70126-s003.tif]

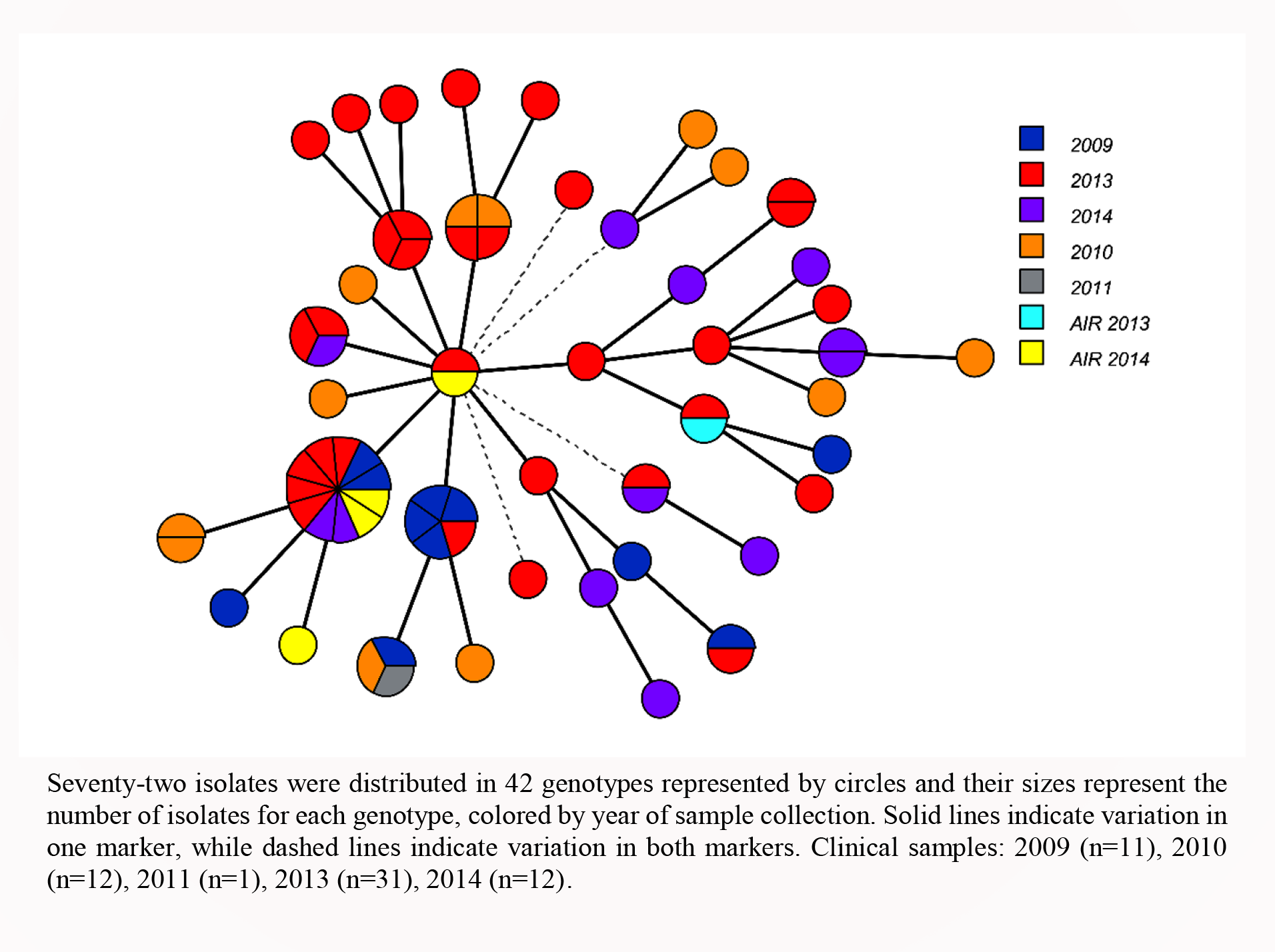

Supplement: Supplementary file 2 — Figure S2: Minimum spanning tree showing the year of collection of clinical and environmental Aspergillus fumigatus isolates genotyped by MC3 and MC5 markers. [file MYC-69-e70126-s002.tif]

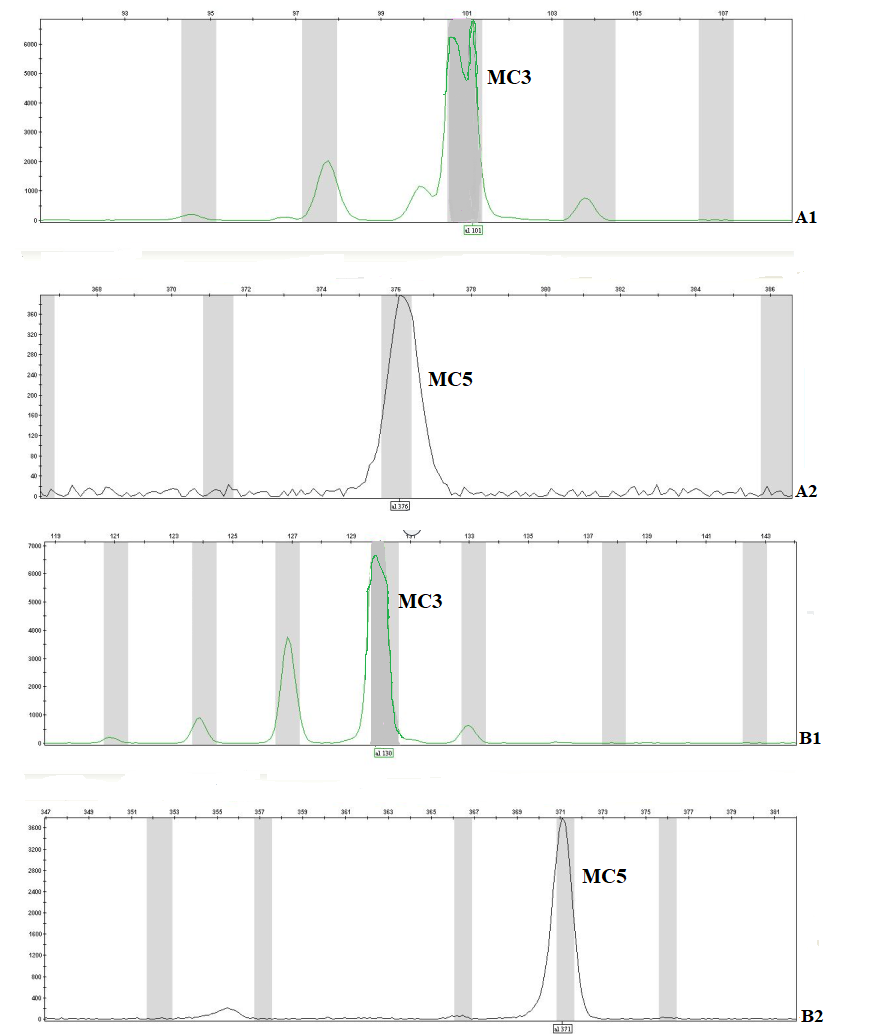

Supplement: Supplementary file 3 — Figure S3: Electrophoretic peaks for MC3 and MC5 markers testing the strains 65 (A1, A2) and 112 (B1, B2) of Aspergillus fumigatus . [file MYC-69-e70126-s005.tif]

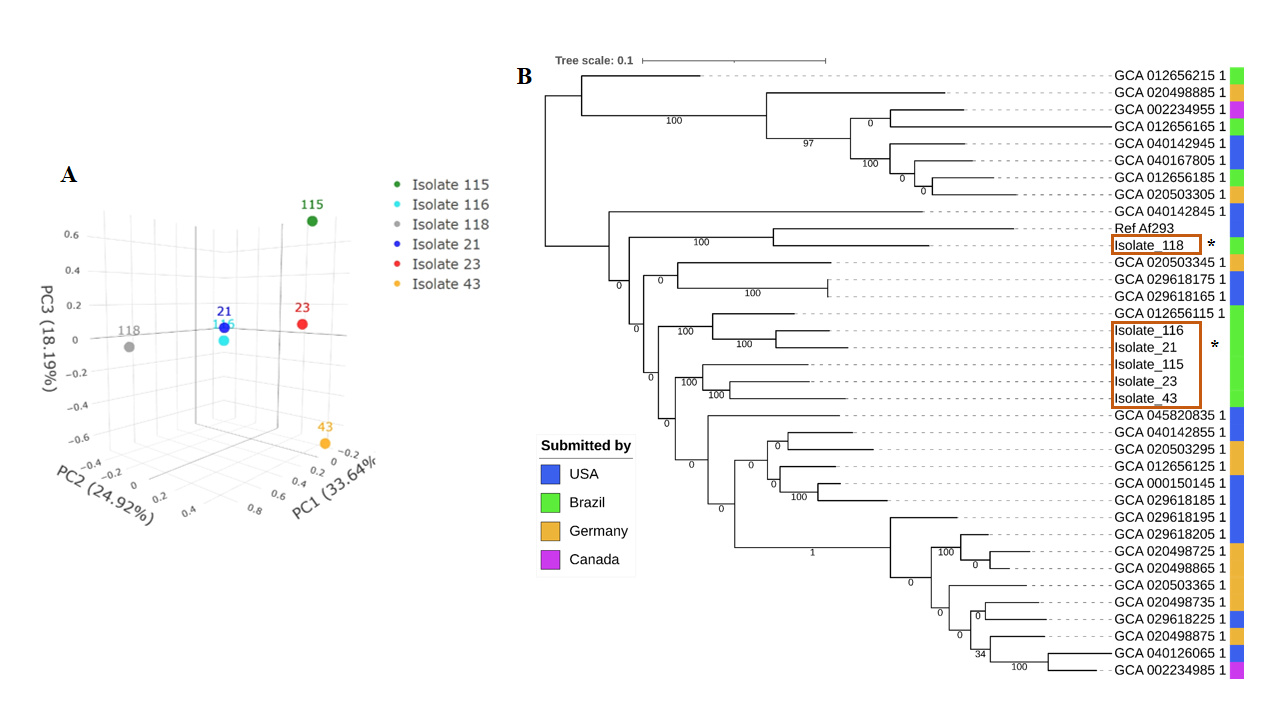

Supplement: Supplementary file 4 — Figure S4: (A) Principal component analysis for the six genomes of this study using the SNPs between the genomes (PC1 represents the first principal component, PC2 the second principal component and PC3 the third principal component), and (B) Phylogenetic tree of Aspergillus fumigatus genomes available at NCBI and six genomes sequenced in this study (marked with *). [file MYC-69-e70126-s001.tif]
