# Supplementary material for: Analysis of Aspergillus spp. Isolates According to Temporal–Spatial, Sociodemographic, and Clinical Variables—Microsatellite Typing of Clinical and Environmental Samples of Aspergillus fumigatus in a University Hospital in Sao Paulo, Brazil
Source: Mycoses. 2026 Jan 17;69(1):e70126. doi: 10.1111/myc.70126 (PMC12811795; doi:10.1111/myc.70126)
Supplement: Supplementary file 5 — Table S1: Distribution of 60 patients according to socio‐demographic and clinical variables. [file MYC-69-e70126-s006.docx]

| **Supplementary Table 1**. Distribution of 60 patients according to socio-demographic and clinical variables | | | | | |  |
| --- | --- | --- | --- | --- | --- | --- |
| **Variable** | | **IPA** | | | **Statistical analysis** |  |
|  |  | **NO** | **YES** | **Total** |  |  |
| **Age (years)** | | **(n=46)** | **(n=14)** | **(n=60)** | ¶ Kolmogorov-Smirnov |  |
| Mean±SD | | 45.57±17.07 | 41.33±21.12 | 42.32±20.19 | **0.200** |  |
| Lower-Upper | | 12-73 | 1-86 | 1-86 |  |  |
| **Sex** | |  |  |  | ‡**Chi-square test** |  |
| Female % (n) | | 43.3(26) | 10.0(6) | 53.3(32) | 0.805 |  |
| **Clinical Forms % (n)** | |  |  |  | §**Fisher's exact test** |  |
| CO | | 61.7(37) | 0(0) | 61.7(37) | **0.000** |  |
| IPA | | 0(0) | 23.3(14) | 23.3(14) |  |  |
| CPSA | | 15.0(9) | 0(0) | 15.0 (9) |  |  |
| **Comorbidities % (n)** | |  |  |  | §**Fisher's exact test** |  |
| Cystic fibrosis or bronchiectasis | | 24.5(13) | 3.8(2) | 28.3(15) | **0.204** |  |
| Transplantation | | 22.6(12) | 5.7(3) | 28.3(15) |  |  |
| Tuberculosis | | 13.2(7) | 0(0) | 13.2(7) |  |  |
| Leukemia, Lymphoma, Multiple Myeloma | | 3.8(2) | 5.7(3) | 9.4(5) |  |  |
| Chronic Respiratory Diseases | | 3.8(2) | 0(0) | 3.8(2) |  |  |
| Others | | 13.2(7) | 3.8(2) | 17.0(9) |  |  |
| **Origin clinics % (n)** | |  |  |  | §**Fisher's exact test** |  |
| PULMO | | 53.3(32) | 6.7(4) | 60.0(36) | **0.000** |  |
| TMO/HEMATO | | 3.3(2) | 15.0(9) | 18.3(11) |  |  |
| Others | | 20.0(12) | 1.7(1) | 21.7(13) |  |  |
| **Clinical samples % (n)** | |  |  |  | §**Fisher's exact test** |  |
| Bronchoalveolar lavage | | 26.7(16) | 13.3(8) | 40.0(24) | **0.273** |  |
| Sputum/tracheal secretion | | 36.7(22) | 5.0(3) | 41.7(25) |  |  |
| Biopsy | | 3.3(2) | 1.7(1) | 5.0(3) |  |  |
| Others | | 10.0(6) | 3.3(2) | 13.3(8) |  |  |
| **Semester/year** | |  |  |  | §**Fisher's exact test** |  |
| 2009-1 | | 1.7(1) | 1.7(1) | 3.3(2) | **0.906** |  |
| 2009-2 | | 8.3(5) | 1.7(1) | 10.0(6) |  |  |
| 2010-1 | | 6.7(4) | 1.7(1) | 8.3(5) |  |  |
| 2010-2 | | 5.0(3) | 1.7(1) | 6.7(4) |  |  |
| 2011-1 | | 1.7(1) | 0(0) | 1.7(1) |  |  |
| 2013-1 | | 18.3(11) | 3.3(2) | 21.7(13) |  |  |
| 2013-2 | | 18.3(11) | 10.0(6) | 28.3(17) |  |  |
| 2014-1 | | 13.3(8) | 3.3(2) | 16.7(10) |  |  |
| 2014-2 | | 3.3(2) | 0(0) | 3.3(2) |  |  |
| **Seasons** | |  |  |  | §**Fisher's exact test** |  |
| summer | | 13.3(8) | 5.0(3) | 18.3(11) | **0.866** |  |
| autumn | | 25.0(15) | 5.0(3) | 30.0(18) |  |  |
| inverno | | 21.7(13) | 6.7(4) | 28.3(17) |  |  |
| spring | | 16.7(10) | 6.7(4) | 23.3(14) |  |  |

CO – Colonised; IPA– Invasive Pulmonary Aspergillosis; CPSA = CPA – Chronic Pulmonary Aspergillosis + Subcutaneous Aspergillosis; BMT–Hemato - Bone Marrow Transplantation-Hematology; PULMO= Pulmonology; Others: Infectology, Dermatology, Neurology, Pediatrics, Cancer Institute. Total number of clinical samples = 60. Missing values for comorbidities (NO IPA= 3, IPA = 4). Statistics: ‡ Chi-square test; §Fisher´s Exact test; ¶Kolmogorov-Smirnov test
