# Supplementary material for: Analysis of Aspergillus spp. Isolates According to Temporal–Spatial, Sociodemographic, and Clinical Variables—Microsatellite Typing of Clinical and Environmental Samples of Aspergillus fumigatus in a University Hospital in Sao Paulo, Brazil
Source: Mycoses. 2026 Jan 17;69(1):e70126. doi: 10.1111/myc.70126 (PMC12811795; doi:10.1111/myc.70126)
Supplement: Supplementary file 6 — Table S2: Number of repeats in markers MC3 and MC5 for each genotype based on raw sequencing data. [file MYC-69-e70126-s004.docx]

**Supplementary Table 2.** Number of repeats in markers MC3 and MC5 for each genotype based on raw sequencing data.

| **Genotype** | **MC3** | **MC5** |
| --- | --- | --- |
| 1 | (TAC)14 | (TTTAT)11 TTCAT (TTTAT)3 (TTAT)3 |
| 2 | (TAC)14 | (TTTAT)11 TTAT (TTTAT)3 (TTAT)3 |
| 3 | (TAC)14 | (TTTAT)12 TTCAT (TTTAT)3 (TTAT)3 |
| 4 | (TAC)15 | (TTTAT)11 TTCAT (TTTAT)3 (TTAT)3 |
| 5 | (TAC)13 | (TTTAT)13 TTCAT (TTTAT)3 (TTAT)3 |
| 6 | (TAC)16 | (TTTAT)12 TTCAT (TTTAT)3 (TTAT)3 |
| 7 | (TAC)17 | (TTTAT)12 TTCAT (TTTAT)3 (TTAT)3 |
| 8 | (TAC)15 | (TTTAT)13 TTAT (TTTAT)3 (TTAT)3 |
| 9 | (TAC)14 | (TTTAT)13 (TTAT)2 |
| 10 | (TAC)13 | (TTTAT)11 (TTAT)5 |
| 11 | (TAC)12 | (TTTAT)8 (TTAT)5 |
| 12 | (TAC)13 | (TTTAT)8 (TTAT)4 |
| 13 | (TAC)15 | (TTTAT)9 (TTAT)2 |
| 14 | (TAC)7 | (TTTAT)11 TTCAT (TTTAT)3 (TTAT)3 |
| 15 | (TAC)8 | (TTTAT)11 TTCAT (TTTAT)3 (TTAT)5 |
| 16 | (TAC)8 | (TTTAT)10 (TTAT)5 |
| 17 | (TAC)11 | (TTTAT)15 TTAT (TTTAT)3 (TTAT)3 |
| 18 | (TAC)9 | (TTTAT)15 TTAT (TTTAT)3 (TTAT)3 |
| 19 | (TAC)7 | (TTTAT)14 TTCAT (TTTAT)3 (TTAT)5 |
| 20 | (TAC)7 | (TTTAT)9 (TTAT)2 |
| 21 | (TAC)24 | (TTTAT)11 TTCAT (TTTAT)3 (TTAT)3 |
| 22 | (TAC)23 | (TTTAT)11 TTCAT (TTTAT)3 (TTAT)3 |
| 23 | (TAC)22 | (TTTAT)13 (TTAT)2 |
| 24 | (TAC)20 | (TTTAT)12 TTCAT (TTTAT)3 (TTAT)3 |
| 25 | (TAC)19 | (TTTAT)12 TTCAT (TTTAT)3 (TTAT)3 |
| 26 | (TAC)19 | (TTTAT)11 TTCAT (TTTAT)3 (TTAT)3 |
| 27 | (TAC)19 | (TTTAT)11 TTAT (TTTAT)3 (TTAT)3 |
| 28 | (TAC)20 | (TTTAT)11 TTCAT (TTTAT)3 (TTAT)3 |
| 29 | (TAC)22 | (TTTAT)15 TTAT (TTTAT)3 (TTAT)3 |
| 30 | (TAC)22 | (TTTAT)14 TTCAT (TTTAT)3 (TTAT)5 |
| 31 | (TAC)21 | (TTTAT)15 TTAT (TTTAT)3 (TTAT)3 |
| 32 | (TAC)23 | (TTTAT)14 TTCAT (TTTAT)3 (TTAT)5 |
| 33 | (TAC)23 | (TTTAT)15 TTAT (TTTAT)3 (TTAT)3 |
| 34 | (TAC)23 | (TTTAT)14 TTAT (TTTAT)3 (TTAT)3 |
| 35 | (TAC)21 | (TTTAT)14 TTAT (TTTAT)3 (TTAT)3 |
| 36 | (TAC)20 | (TTTAT)11 TTCAT (TTTAT)3 (TTAT)5 |
| 37 | (TAC)19 | (TTTAT)14 TTAT (TTTAT)3 (TTAT)3 |
| 38 | (TAC)17 | (TTTAT)16 TTCAT (TTTAT)3 (TTAT)5 |
| 39 | (TAC18 | (TTTAT)15 TTCAT (TTTAT)3 (TTAT)5 |
| 40 | (TAC)16 | (TTTAT)14 TTCAT (TTTAT)3 (TTAT)5 |
| 41 | (TAC)15 | (TTTAT)28 (TTAT)5 |
| 42 | (TAC)16 | (TTTAT)27 (TTAT)5 |
